# Supplementary figures and images for: Tardigrade Community Microbiomes in North American Orchards Include Putative Endosymbionts and Plant Pathogens
Source: Front Microbiol. 2022 Jul 18;13:866930. doi: 10.3389/fmicb.2022.866930 (PMC9340075; doi:10.3389/fmicb.2022.866930)

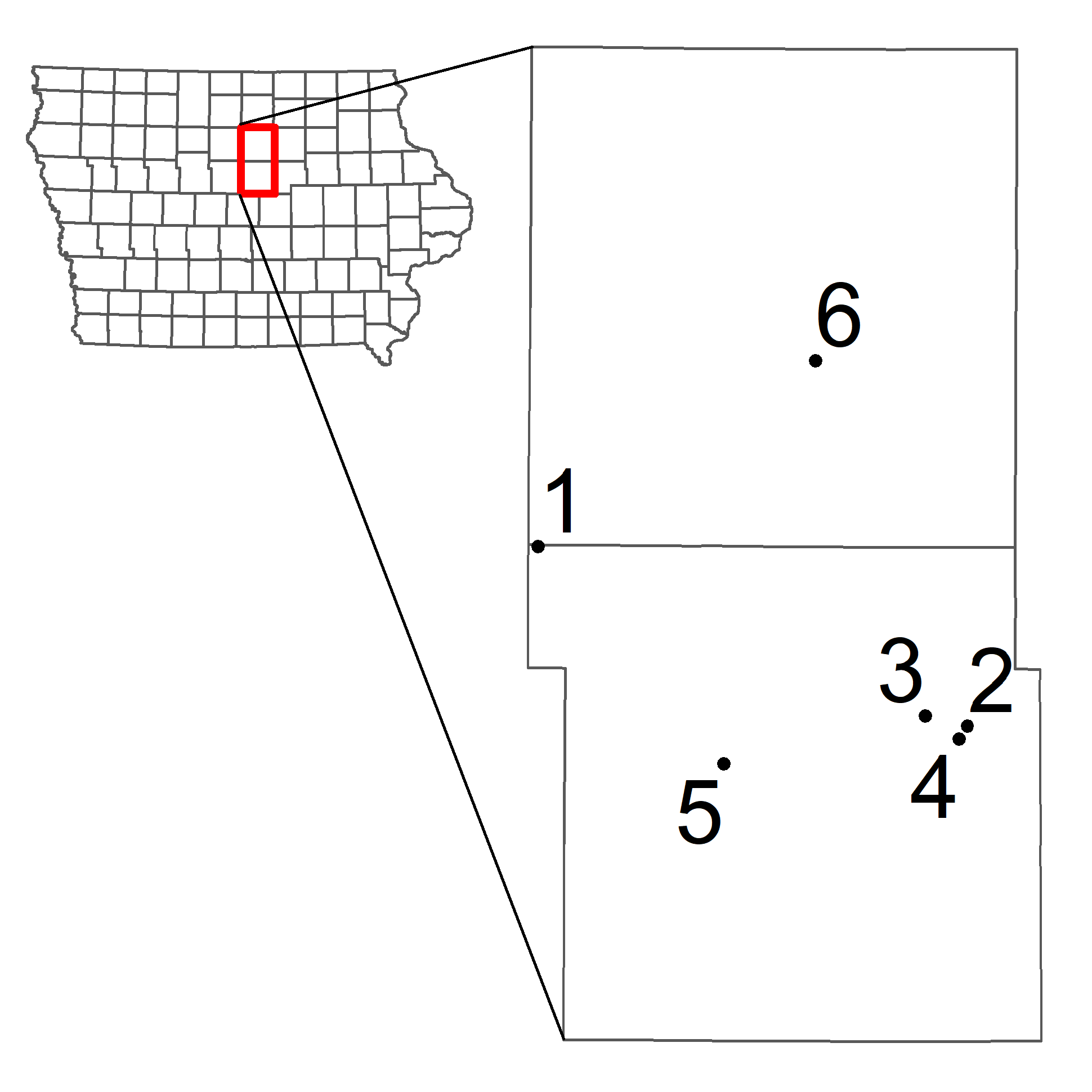

Supplement: Supplementary Figure S1 — Collection locations map. The map of Iowa, United States shows the sampled counties outlined in red. The inset shows collection sites within Hardin and Franklin counties identified by location number. [file Image_1.TIF]

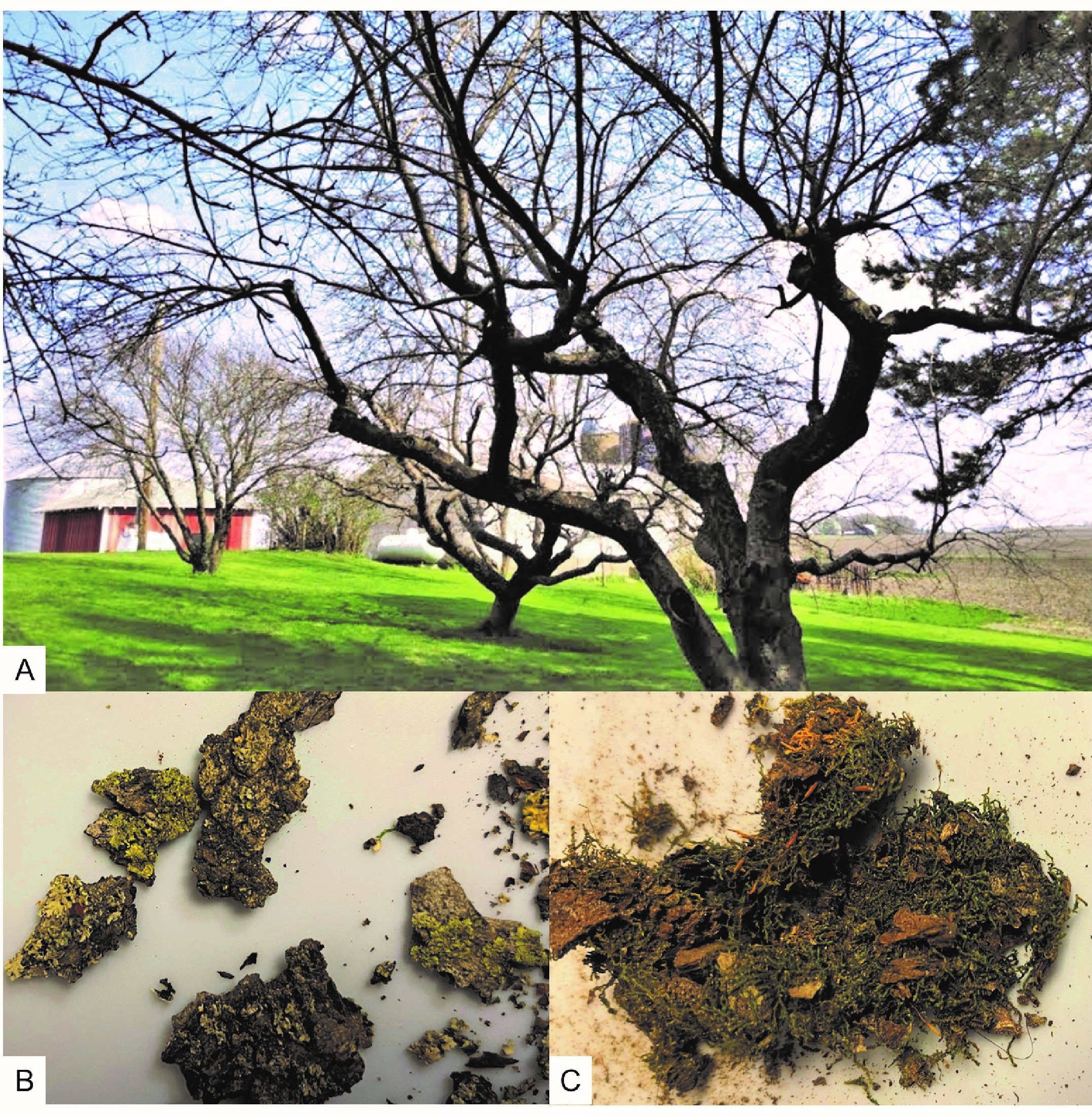

Supplement: Supplementary Figure S2 — Representative images of sampled trees and substrates. (A) Tree 2, Location 1 (B) Lichen collected from Tree 2, Location 1 (C) Moss collected from Tree 1, Location 2. [file Image_2.JPEG]

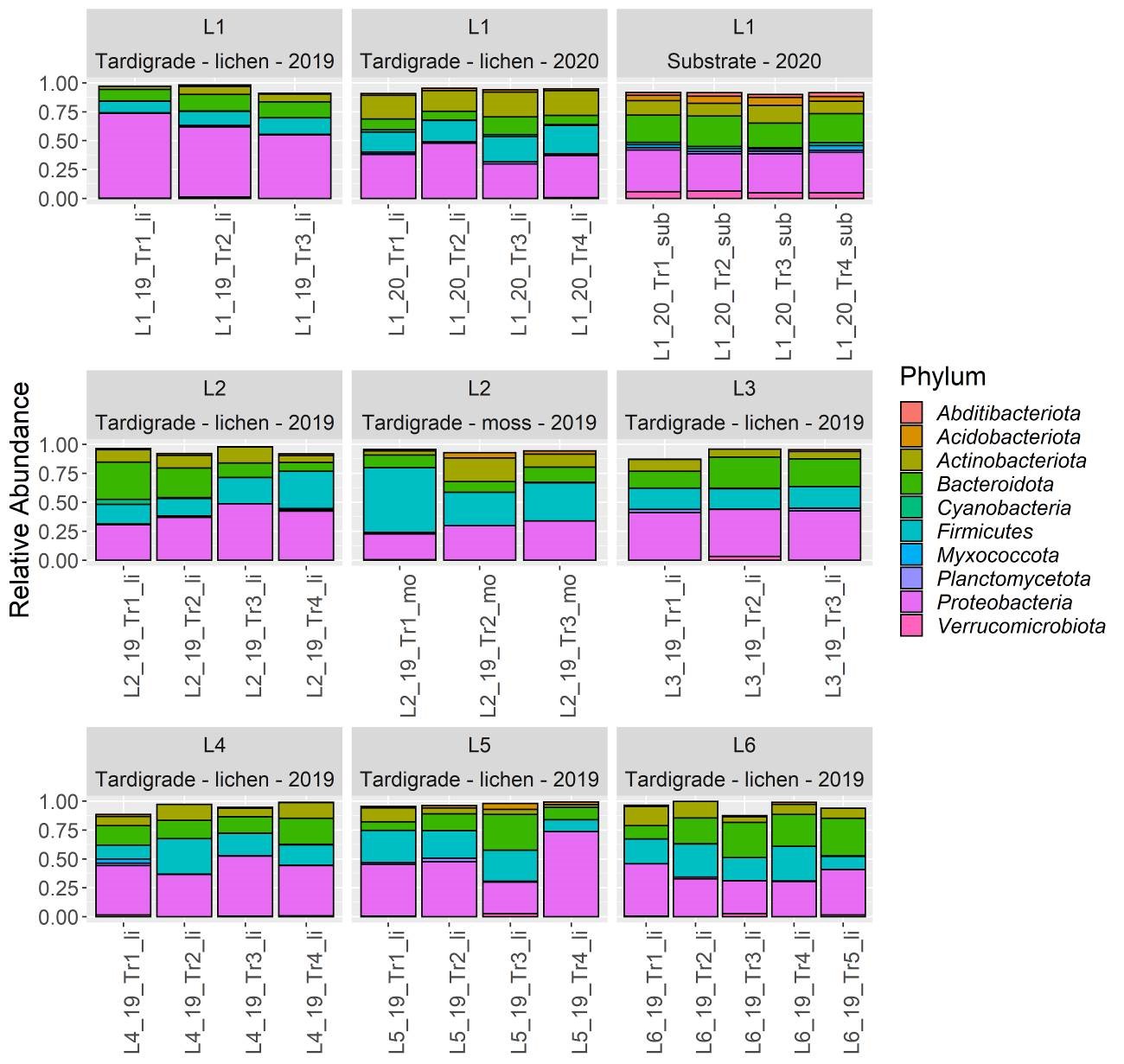

Supplement: Supplementary Figure S3 — Relative abundance of top 10 identifiable phyla shown across all samples. Location names (Location 1 through Location 6) are abbreviated as L1 through L6. [file Image_3.jpeg]

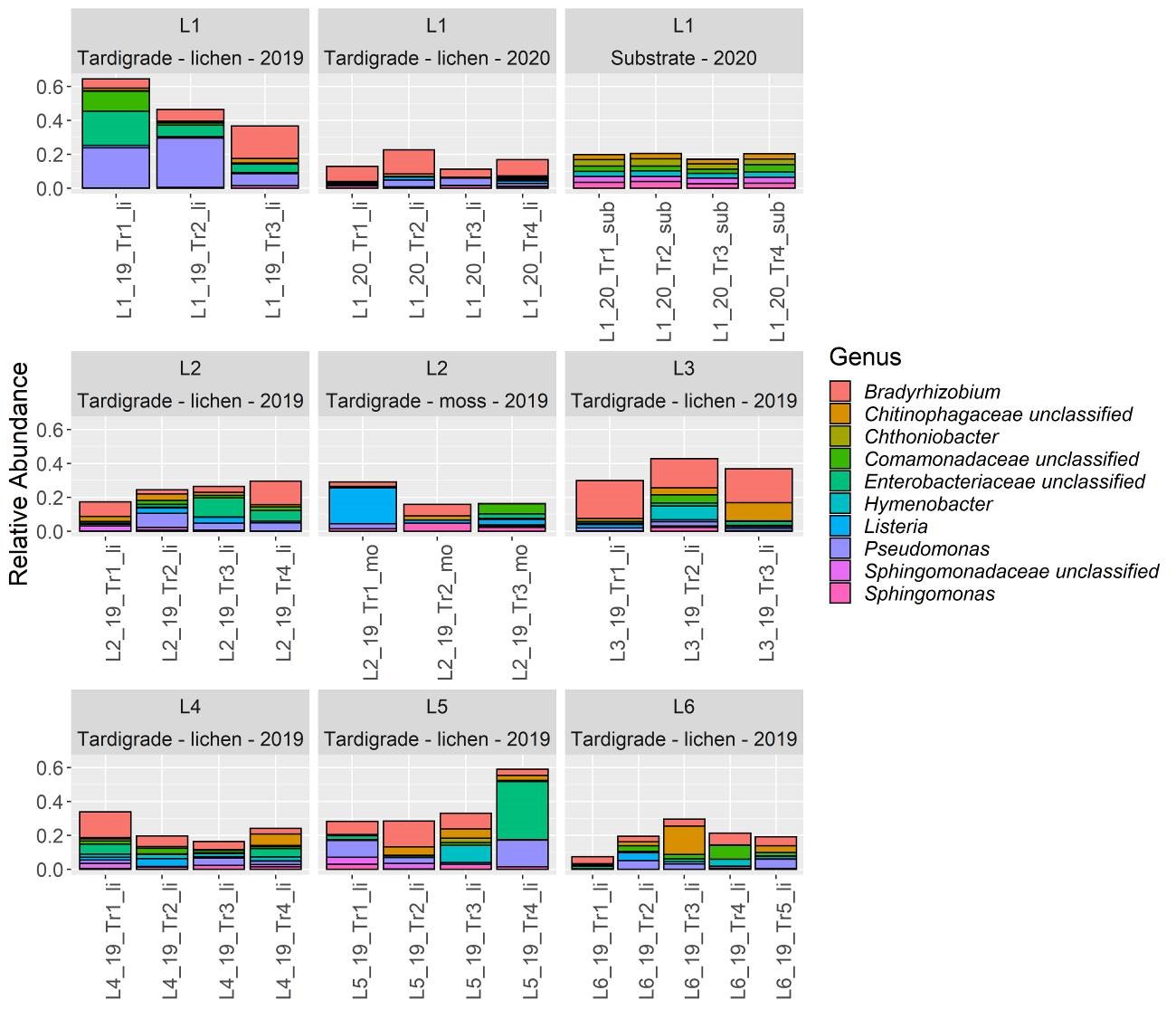

Supplement: Supplementary Figure S4 — Relative abundance of top 10 genera (identifiable at least to family level) shown across all samples. Location names (Location 1 through Location 6) are abbreviated as L1 through L6. [file Image_4.JPEG]

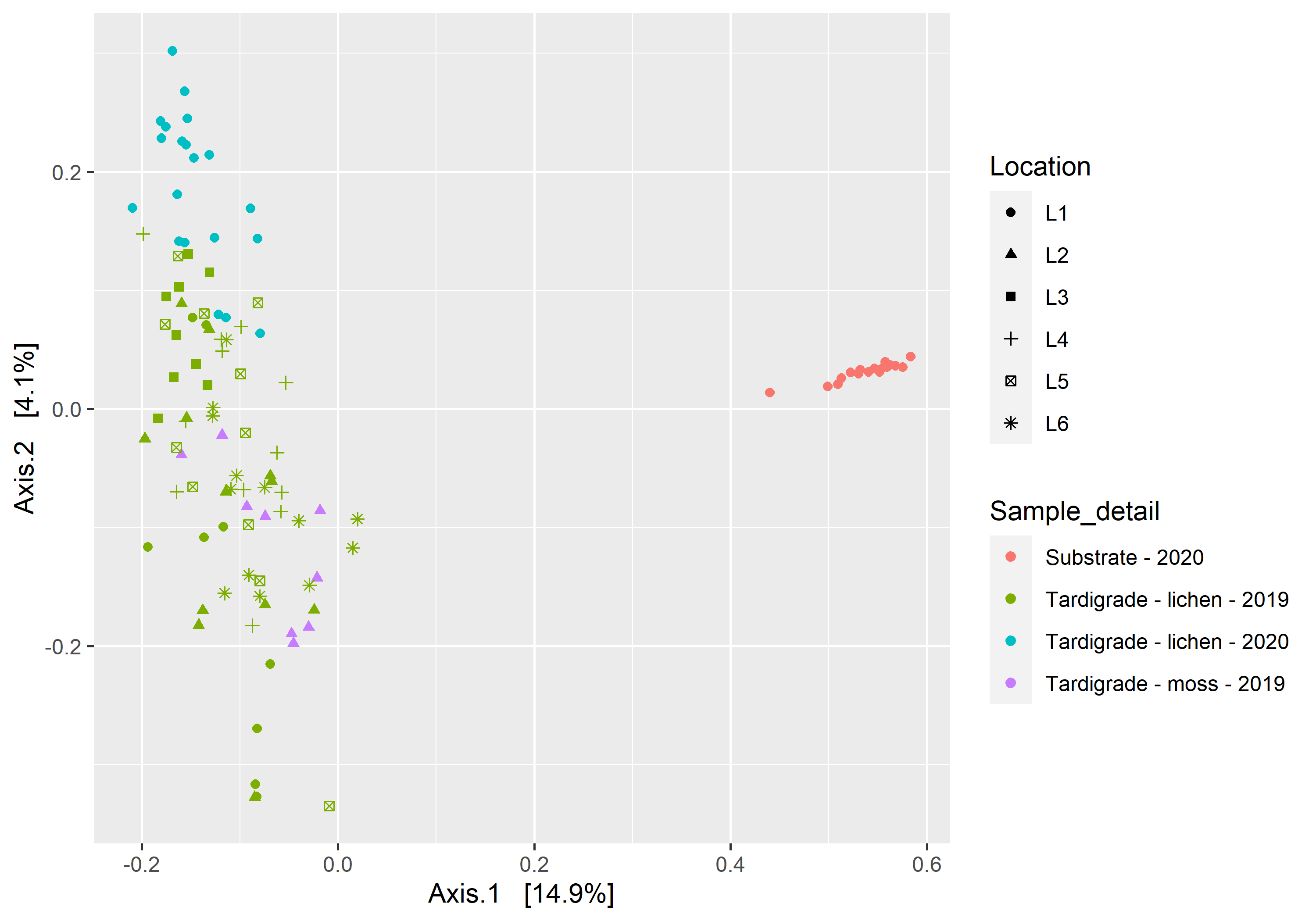

Supplement: Supplementary Figure S5 — Principal Coordinates Analysis of all samples based on Bray-Curtis distance. Location names (Location 1 through Location 6) are abbreviated as L1 through L6. Substrate samples are clearly separated from the tardigrade samples along Axis 1. On Axis 2, samples from 2020 are generally clustered away from 2019 samples. Samples from different locations and from lichen and moss overlap. [file Image_5.tif]
